# Global burden of type 2 diabetes attributable to non-high body mass index from 1990 to 2019

**DOI:** 10.1186/s12889-023-15585-z

**Published:** 2023-07-12

**Authors:** Jingjing Wu, Zeying Feng, Jingwen Duan, Yalan Li, Peizhi Deng, Jie Wang, Yiping Yang, Changjiang Meng, Wei Wang, Anli Wang, Jiangang Wang

**Affiliations:** 1 Health Management Center, The Third Xiangya Hospital, Central South University, 138 Tongzipo Road, Yuelu District, Changsha City, Hunan Province 410013 China; 2 Clinical Research Center, Central South University, Changsha, Hunan China; 3 Clinical Trial Institution Office, Liuzhou Hospital of Guangzhou Women and Children’s Medical Center, No. 50 Boyuan Avenue, Liuzhou City, Guangxi Province 545001 China; 4 Information Center of The Third Xiangya Hospital, Central South University, 138 Tongzipo Road, Yuelu District, Changsha City, Hunan Province 410013 China

**Keywords:** Type 2 diabetes mellitus, Body mass index, Global burden of disease

## Abstract

**Background:**

The prevalence of type 2 diabetes mellitus (T2DM) currently was increased in some countries of the world like China. However, the epidemiological trends of T2DM attributable to non-high body mass index (BMI) remain unclear. Thus, we aimed to describe the burden of T2DM attributable to non-high BMI.

**Methods:**

To estimate the burden of T2DM attributable to non-high BMI, data from the Global Burden of Disease Study 2019 were used to calculate the deaths and disability-adjusted life years (DALYs) by age, sex, year, and location. The estimated annual percentage change (EAPC) was applied in the analysis of temporal trends in T2DM from 1990 to 2019.

**Results:**

Globally in 2019, the number of death cases and DALYs of T2DM attributable to non-high BMI accounted for 57.9% and 48.1% of T2DM-death from all risks, respectively. Asia accounted for 59.5% and 63.6% of the global non-high-BMI-related death cases and DALYs of T2DM in 2019, respectively. From 1990 to 2019, regions in the low-income experienced a rise in DALYs attributable to non-high BMI. As compared to other age groups, older participants had higher deaths and DALYs of T2DM attributable to non-high BMI. The death and DALY rates of T2DM due to non-high BMI were higher in males and people in regions with low socio-demographic index (SDI) countries.

**Conclusions:**

The burden of T2DM attributable to non-high BMI is higher in the elderly and in people in regions with low- and middle-SDI, resulting in a substantial burden on human health and the social cost of healthcare.

**Supplementary Information:**

The online version contains supplementary material available at 10.1186/s12889-023-15585-z.

## Introduction

Diabetes poses a serious public health issue with considerable consequences for human life and health expenditures. According to International Diabetes Federation, the number of adults with diabetes was estimated at 536.6 million people in 2021 and will rise to more than 783.2 million by 2045 [1]. Over 90% of all diabetes cases are type 2 diabetes mellitus (T2DM), which is the fifth-ranking cause of mortality among individuals aged 50 to 74 [2, 3]. Thus, identifying the risk factors of T2DM is essential for the early control and prevention of T2DM.

High body mass index (BMI) is the main modifiable risk factor of T2DM and the global burden of T2DM due to high BMI has been studied extensively [4, 5]. The prevalence of T2DM currently has been reported in non-high BMI (BMI < 25 kg/m^2^) individuals and appeared racial differences. For instance, studies found that the prevalence of T2DM among non-high BMI adults was 7.8% in China and 4.3% in the United States [6, 7]. In addition, individuals from Asia and Africa had, in the case of equivalent incidence rate of T2DM, much lower BMI levels compared with individuals in other race/ethnicity groups [8, 9]. In fact, the prevalence of abdominal obesity was higher among patients who were not categorized as obese based on BMI criteria, compared to those with normal waist circumference [10]. Studies have shown that individuals with non-high BMI, but with metabolic syndrome (MetS) or lean non-alcoholic fatty liver disease (NAFLD), had an increased risk of T2DM than individuals without MetS or NAFLD, whereas a considerable proportion of overweight and obese individuals were metabolically healthy [11–13]. Since BMI does not fully capture body composition, especially visceral adipose tissue, standard BMI classifications do not fully reflect the distribution of body fat.

Currently, diabetes prevention measures are mostly aimed at individuals who are overweight or obese, as determined by their BMI [14, 15]. Participants with a low BMI were less likely to receive medical therapies to reduce their health risks. In addition, the burden of T2DM attributable to non-high BMI continues to receive insufficient attention. Therefore, it is essential to understand the current burden of non-high BMI-associated T2DM in different regions of the world and to estimate trends in this burden over time. Therefore, we aimed to analyze the burden of T2DM attributable to non-high BMI in 204 countries. In this context, we assessed the age-standardized mortality and disability-adjusted life-year (DALY) rates and temporal trends of T2DM attributable to non-high BMI in these regions.

## Materials & methods

### Data sources

The Global Burden of Disease (GBD) Study 2019 provided global and systematic assessments of 369 diseases and injuries, 286 causes of mortality, and 87 risk factors across 204 countries and territories. According to epidemiological similarity and geographic proximity, the 204 countries and territories were divided into 21 regions. Detailed methodologies of GBD 2019 and the comparative risk assessment have been reported previously. In this study, we downloaded counts and rates of mortality and DALYs from the Global Health Data Exchange (GHDEx) website (https://vizhub.healthdata.org/gbd-results/) for T2DM as well as a relative burden due to high BMI. Estimates were given 95% uncertain intervals (UIs), which included sources of uncertainty such as measurement error, possible biases, and modeling. Health estimates reported in this study comply with the Guidelines for Accurate and Transparent Health Estimates Reporting (GATHER). Because GBD 2019 uses deidentified summary data, informed consent was waived by the University of Washington Institutional Review Board [16].

DALYs was the summation of the years lived with disability (YLDs), according to standardized disability weights for each health state, and the years of life lost (YLLs), according to a reference maximum observed life expectancy. The DALYs is a summary measure that quantifies the overall burden of disease. One DALY can be regarded as the loss of 1 year in full health life spans. The Socio-demographic Index (SDI) is a composite indicator of a geographical location’s development status which was calculated by total fertility rates among females under the age of 25, mean educational attainment for individuals aged 15 years and older, and lag distributed income per capita. SDI is expressed from 0 to 1, where 0 represents the lowest level of development, and 1 represents the highest level of development. Detailed methods for SDI were provided in Additional file 1. Based on the SDI quintiles, the 204 countries and regions were divided into five groups: low, low-middle, middle, high-middle, and high SDI regions.

### Statistical analysis

To quantify the attributable burdens of T2DM due to non-high BMI, we defined the burdens of T2DM due to non-high BMI by using the total burden of T2DM minus the burden of T2DM due to high BMI. All estimates of the number of deaths or DALYs, the age-standardized mortality rate (ASMR), age-standardized DALY rate (ASDR), and percent change were reported overall or by age, sex, year, and location.

Age-standardized rates (ASR) were calculated by standardization to the global age structure which was used to eliminate the interference caused by differences in population structures when comparing different populations or the same population in different periods. To estimate time trends of ASMR and ASDR for T2DM due to non-high BMI from 1990 to 2019, we calculated the estimated annual percentage change (EAPC). EAPC is a widely used index that describes the trend of ASR over a specific time, which was calculated by fitting a linear regression line to the natural logarithm of the rates. The EAPC and its 95% confidence interval (CI) were estimated using the linear regression model. The formulas were as follows:

$$y=\alpha +\beta x+\varepsilon$$

$$\mathrm{EAPC}=100*(\mathrm{exp}(\upbeta ) -1)$$

where $y$ means ln(ASR) value, *x* refers to the calendar year, $\beta$ means the annual change in ln(ASR), and $\varepsilon$ means the error term. Positive EAPC here represents an increasing trend of ASMR or ASDR, and negative EAPC represents a decreasing trend of ASMR or ASDR. Otherwise, the burden of T2DM attributable to non-high BMI is regarded as stable. Finally, to indicate whether the level of SDI in a country is a major determinant of the rate of deaths and DALYs from T2DM, we examined the relationship between SDI and the burden of T2DM attributable to non-high BMI from 1990 to 2019 in 21 regions. All statistical analyses were performed using R 4.1.2 version and Microsoft Excel.

## Results

### Overview

Worldwide in 2019, there were 853,439 death cases of T2DM attributable to non-high BMI accounting for 57.9% of total death of T2DM (Additional file 2 Table S1). Furthermore, the global DALYs of T2DM attributable to non-high BMI is 3.2 million, accounting for 48.1% of T2DM-DALYs from all risks (Additional file 2 Table S2). Although high BMI is one of the top risk factors for T2DM, non-high BMI was also an important contributor to T2DM in some countries in the world in 2019. Among 21 world regions, of the global non-high-BMI-related death cases and DALYs to T2DM in 2019, 59.5% and 63.6% occurred in 5 Asia regions, accounting for 507,960 death cases and 2.0 million DALYs, respectively. At the national level, following India, mainland China has the highest burden of T2DM due to non-high BMI, contributing to 14.2% death cases and 18.4% DALYs of total death and DALYs globally in 2019 non-high-BMI-attributed T2DM.

### The burden of T2DM attributable to non-high BMI in 2019

**Fig. 1:**
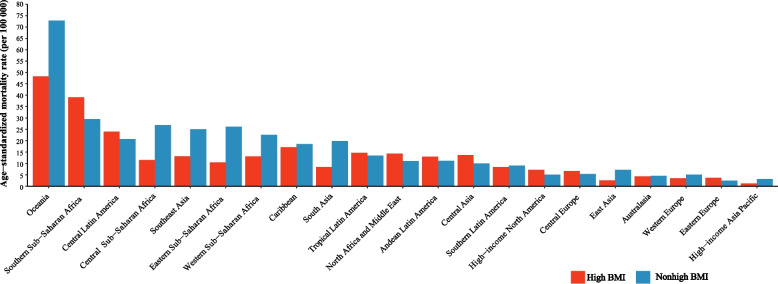
ASMR of T2DM attributable to non-high BMI and high BMI for 21 regions, in 2019. Abbreviations: ASMR, age-standardized mortality rate; T2DM, type 2 Diabetes Mellitus; BMI, body mass index

In 2019, the ASMR and ASDR of T2DM attributable to non-high BMI were 10.92/100,000 and 390.49/100,000 in the world’s population, respectively (Additional file 2 Table S3 and Table S4). Across the 21 GBD regions, compared to high-BMI-related ASMR of T2DM, the ASMR of T2DM attributable to non-high BMI was higher significantly in Oceania (72.72/100,000 vs 48.3/100,000), Central sub-Saharan Africa (26.84/100,000 vs 11.45/100,000), Eastern sub-Saharan Africa (26.17/100,000 vs 10.35/100,000), Southeast Asia (24.96/100,000 vs 13.1/100,000), Western sub-Saharan Africa (22.53/100,000 vs 13.06/100,000) and South Asia (19.74/100,000 vs 8.4/100,000) which were located in low and low-middle SDI region (Additional file 2 Table S3, Fig. 1). Similar patterns were observed for DALYs, ASDR of T2DM attributable to non-high BMI was higher significantly in Oceania (1902.15/100,000 vs 1801.28/100,000), Central sub-Saharan Africa (779.38/100,000 vs 485.7/100,000), Southeast Asia (718.20/100,000 vs 555.22/100,000), Eastern sub-Saharan Africa (643.44/100,000 vs 383.38/100,000), South Asia (620.88/100,000 vs 428.83/100,000) and Western sub-Saharan Africa (532.07/100,000 vs 462.75/100,000) (Additional file 2 Table S4, Additional file 1 Fig. S1).

**Fig. 2:**
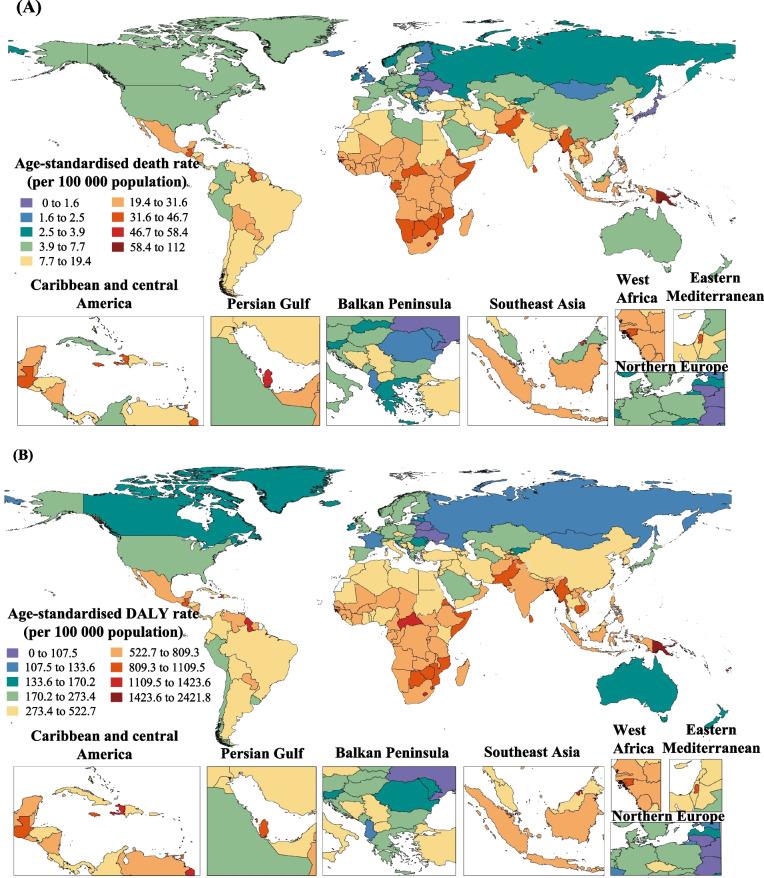
Global burden of T2DM attributable to non-high BMI in 204 countries and territories. **A** Age-standardized mortality rate (ASMR) in 2019; **B** Age-standardized disability-adjusted life-year rate (ASDR) in 2019. Abbreviations: T2DM, type 2 Diabetes Mellitus; BMI, body mass index

The global map of ASMR and ASDR of T2DM attributable to non-high BMI at the country levels exists considerable heterogeneity (Fig. 2). The ASMR of T2DM attributable to non-high BMI varied by 110.9 times, ranging from 0.95/100,000 in Belarus to 105.32 /100,000 in Fiji (Fig. 2A). The national ASDR of T2DM attributable to non-high BMI varied by 20.8 times, ranging from 84.82/100000 in Belarus to 1766.57/100000 in Kiribati (Fig. 2B).

### Temporal trends of T2DM burdens attributable to non-high BMI from 1990 to 2019

Between 1990 and 2019, the global ASMR and ASDR of T2DM due to non-high BMI were slightly decreased by -0.33% and -0.18%, respectively (Additional file 2 Table S3 and Table S4). Despite the decreases, the total death cases and DALYs of T2DM attributable to non-high BMI almost doubled over the study period, increasing by 105.45% and 98.15%, respectively (Additional file 2 Table S1 and Table S2).

The most pronounced growth in ASMR and ASDR of T2DM due to non-high BMI both in Central Asia (2.63% in death and 1.96% in DALYs) over the study period, while the largest decline in High-income Asia Pacific (-2.59% in death) and Tropical Latin America (-1.5% in DALYs), respectively (Additional file 2 Table S3 and Table S4).

In all 204 countries and territories, the ASMR trend of T2DM attributable to non-high BMI ranged from Singapore (EAPC = -7.69%, 95% CI -8.74 ~ -6.64) to Mauritius (EAPC = 4.59%, 95% CI 3.62 ~ 5.58) (Additional file 2 Table S3). The national ASDR trend of T2DM attributable to non-high BMI ranged from Singapore (EAPC = -3.25%, 95% CI -3.52 ~ -2.98) to Uzbekistan (EAPC = 3.34%, 95% CI 2.84 ~ 3.84) (Additional file 2 Table S4).

### Age- and sex-specific T2DM burden attributable to non-high BMI

**Fig. 3:**
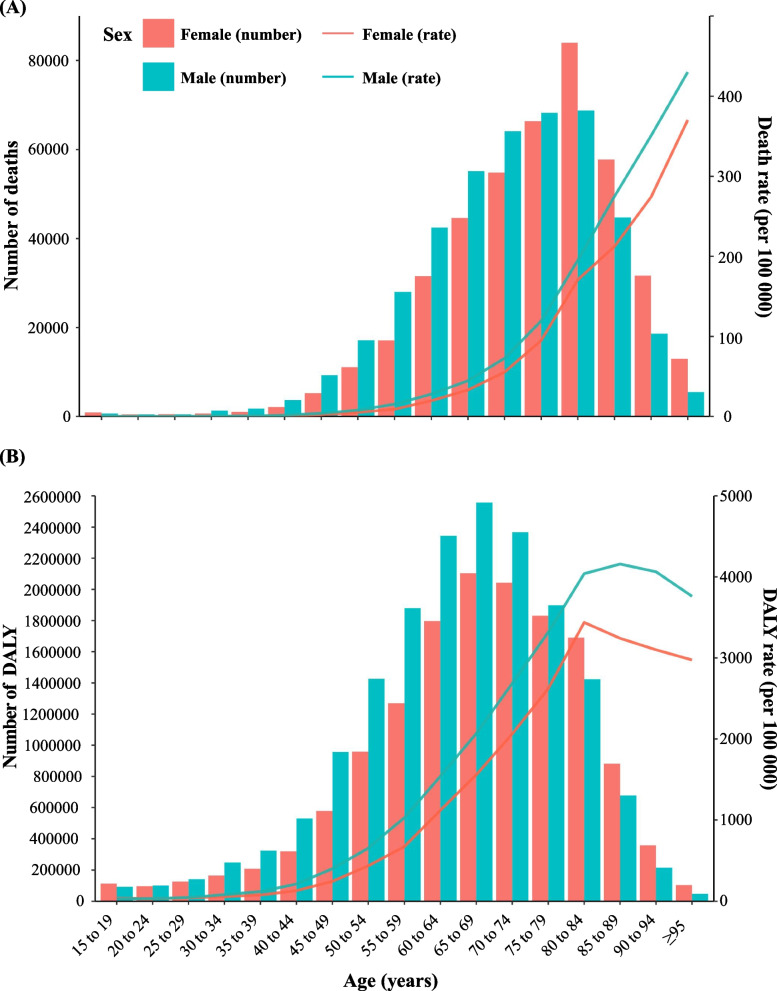
Age-specific numbers and rates of deaths and DALYs for T2DM attributable to non-high BMI. **A** Age-specific numbers and rates of deaths by sex in 2019. **B** Age-specific numbers and rates of DALYs by sex in 2019. Abbreviations: DALYs, disease-adjusted life-years; T2DM, type 2 Diabetes Mellitus; BMI, body mass index

By sex, the non-high-BMI-attributable deaths and DALYs rates of T2DM were significantly higher in males than females (Fig. 3A and B). By age group, the trends of mortality and DALY rate of T2DM due to non-high BMI were largely consistent, the deaths rate increased by age until age ≥95 years in both sexes, while the DALY rate increased by age until age 85 to 89 years in men and 80 to 84 years in women, thereafter it decreased. The highest number of death of T2DM attributable to non-high BMI occurred in the group aged 80–84 years in males and females, while the highest number of DALYs was observed in the age group 65—69 years in both sexes, respectively.

### Association of T2DM burden attributable to non-high BMI and SDI

**Fig. 4:**
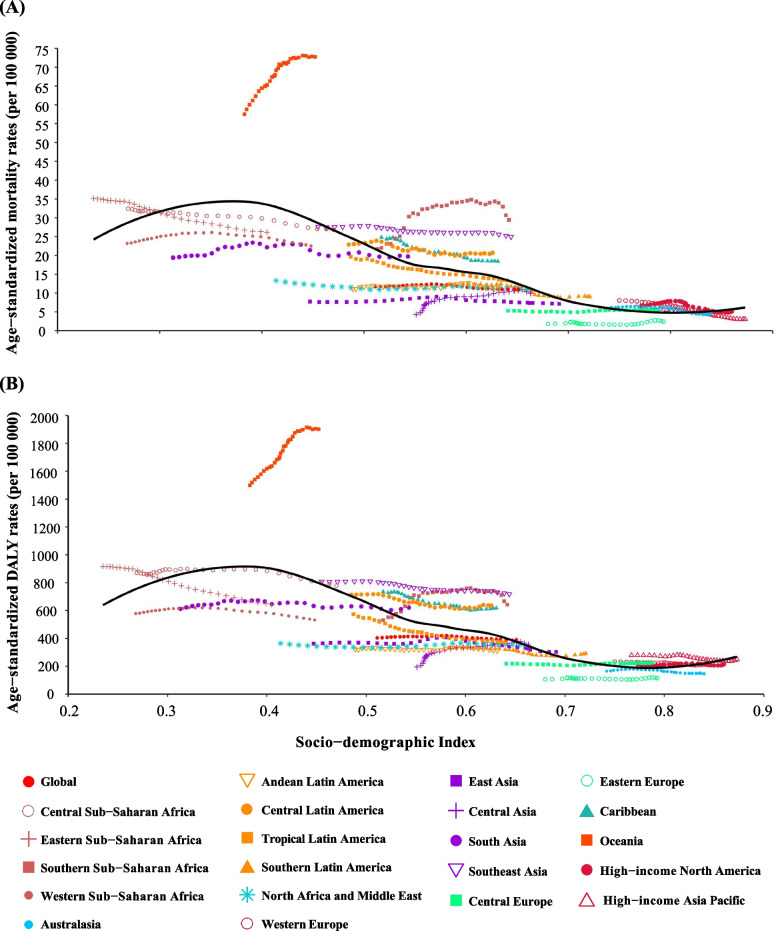
Association between T2DM burden attributable to non-high BMI and SDI for 21 world regions. **A** Age-standardized mortality rate (ASMR) between 1990 and 2019; **B** Age-standardized disability-adjusted life-year rate (ASDR) between 1990 and 2019. Abbreviations**:** BMI, body mass index; SDI, socio-demographic index; T2DM, type 2 Diabetes Mellitus

We illustrated the associations between the ASR and SDI in the 21 regions from 1990 to 2019 (Fig. 4). During the past 30 years, the ASMR and ASDR slightly decreased or remained steady in all regions except Oceania. In Oceania, the observed ASMR and ASDR not only increased over the study period but increased to much higher rates than those that would be expected based solely on SDI. Overall, the ASMR and ASDR of T2DM attributable to non-high BMI both slightly raised with increasing SDI when the SDI was under 0.4 and then showed a roughly decreasing trend with the increase of SDI, indicating the greatest burdens showing in counties with low SDI.

## Discussion

In this study, we estimated the death counts and DALYs and corresponding ASR for T2DM attributable to non-high BMI in 204 countries from 1990 to 2019. Globally, there were almost 853,439 death cases and 3.2 million DALYs which show a highlighted significant, yet under-recognized, global burden of T2DM due to non-high BMI. Owing to their large population sizes, India and China in Asia had the largest portion of both global death cases and DALYs of T2DM attributable to non-high BMI. However, the non-high-BMI-attributed mortality and DALY rates in this region of the globe showed slightly decreasing trends once age standardization was applied. This may be due to the burden of T2DM attributable to non-high BMI increases as the worldwide population ages.

Notably, Central Asia experienced the highest increases in ASMR and ASDR between 1990 and 2019, even though Oceania had the highest ASMR and ASDR rates of T2DM attributable to non-high BMI. Asians had higher levels of postprandial glycemia and lower insulin sensitivity than Whites in response to a realistic carbohydrate load, implying a prevalent genetic predisposition to insulin resistance and diabetes in Asians than whites [17]. The genetic factor influences T2DM mainly by controlling the β-cell dysfunction and body fat distribution. One study documented that KCNJ15, as a susceptibility gene for T2DM, can lead to β-cell dysfunction in Asians [18]. Furthermore, several genetic loci including PAX4, PSMD6, ZFAND3, NID2, and ALDH2, which are associated with insulin secretion, body fat distribution, and β-cell dysfunction, were observed more frequently in East Asians [19]. In addition, studies found that South Asians from UK biobank have a higher number of risk alleles associated with β-cell dysfunction and fewer protective alleles associated with the ability to store extra fat in subcutaneous adipose tissue than white European [20, 21]. The genetic difference could partly explain the differences in β-cell function and body fat distribution and its relationship with T2DM. A growing body of evidence indicated that exposure to environmental pollutants was associated with increased insulin resistance, MetS, and diabetes [22–24]. Most Asian countries, especially India and China, have the worst ambient particulate matter in the world according to data from the World Health Organization (WHO) [25]. The mortality as a cause-specific death rate attributed to fine particulate matter increased during the spread of the unhealthy environment, with China and India accounting for more than half of all deaths due to air pollution in 2015 [26]. Among them, air pollution in Central Asia has become increasingly prevalent, which partly explains the rapid increase in the burden of diabetes in the region [27]. In addition, long-term arsenic exposure has also been shown to be associated with diabetes in studies from Taiwan and Bangladesh [28].

In 2019, we find that the mortality and DALYs rates of T2DM attributable to non-high BMI between males and females were different and more pronounced in men than in women. The reason for this phenomenon is not completely understood. However, except in some parts of the world, the prevalence rate of T2DM in adult populations was found to be significantly higher in males than in females [29, 30]. Moreover, the burden of T2DM attributable to non-high BMI is concentrated in older patients which can be explained by rising life expectancy in many countries and the aging population.

Our findings illustrated that the burden of T2DM attributable to non-high BMI varied substantially across regions, mainly in regions with low and middle SDI. Rapid economic development has led to an increasing burden of diabetes in many parts of the globe. According to IDF, around 12.5% of global health expenditure on diabetes is being consumed in low- and middle-income economies. International Diabetes Federation. IDF Atlas 10th edition. 2021. https://diabetesatlas.org/atlas/tenth-edition. Accessed 5 July 2023. There is a complex non-linear association between ASDR for the 21 world regions and SDI over the 1990 to 2019 period which indicated that the burden of T2DM attributable to non-high BMI cannot be fully explained by income levels.

Obesity is an important risk factor contributing to T2DM, but there are significant differences in reports of BMI-related mortality outcomes among patients with T2DM [31]. Previous studies have reported that there is a nonlinear association between BMI and mortality in people with T2DM like U or J [32–34]. Except for high BMI groups (> 30 kg/m^2^), low- and normal-weight patients with T2DM were associated with higher mortality compared with the reference group (25–29.9 kg/m^2^) [34]. The reason for this phenomenon can be explained by several reasons. (1) Insulin resistance plays an integral role in the pathogenesis of T2DM and also is the core mechanism of sarcopenic obesity, related to visceral fat deposition and reduced muscle mass [35, 36]. People with T2DM can have the significant abdominal circumference, despite the absence of significant obesity as expressed by BMI. Except for over-nutrition, poor nutrition during fetal development and early childhood can promote a fat-preserving or thrifty phenotype which predisposes individuals to insulin resistance and reduced beta cell function [37]. A basic study found that gestational low-protein programming produced a progressively worsening T2DM with the lean phenotype in a rat model [38]. (2) Genetic susceptibility. Single nucleotide polymorphisms representing 36 types 2 diabetes loci, among which 29 had a greater odds ratio in the lean with T2DM compared to obese subsets, indicating lean individuals may have a stronger genetic predisposition to T2DM [39]. (3) Several lifestyle factors, such as lack of exercise, poor diet, smoking, and abstinence, have been reported can increase the risk of T2DM, independently of body weight [40]. (4) Reverse causation may explain the lower risk of mortality among patients being obese compared with participants with normal weight [41]. (5) Patients with high BMI received earlier and better medical treatment than those with normal BMI, which may explain the higher risk of mortality among patients with low BMI levels [42].

The major advantage of our study included that we used the data from GBD estimates, which provide the cause-specific mortality available for each age, sex, year, and location around the world. Further, we estimated the effect of non-high BMI on T2DM and the temporal trend. However, there are several limits to our research. Firstly, although BMI is a universal measure of whole-body fat distribution, there are still presented challenges for BMI in distinguishing between skeletal muscle and body fat and in evaluating abdominal adiposity. Secondly, our study may fail to capture weight loss in some populations, such as those in the later stages of T2DM, when evaluating the burden of diabetes, which may affect the burden of T2DM due to individuals may lose weight both intentionally and unintentionally, while requiring anti-diabetic medications. Thirdly, given the absence of data sources in some locations, GBD estimates depend significantly on the modeling process which may lead to some uncertainty. In addition, due to a lack of sophisticated advanced diagnostic methods, the GBD framework of estimates is prone to underestimate T2DM mortality in low-SDI locations. Finally, the same cutoffs utilized may indicate different levels of obesity in different populations, such as in Asia, including China, and populations at risk or with underlying comorbidities.

## Conclusions

Taken together, using GBD 2019 data, we have produced the increasing global burden of T2DM attributable to non-high BMI between 1990 and 2019. The burden of T2DM due to non-high BMI exposure is higher in the elderly and in populations in regions with low- and middle-SDI, resulting in a substantial burden on human health and the social cost of healthcare. While high BMI is the main driver of mortality for T2DM, the burden of T2DM resulting from non-high BMI also should receive more attention with supportive policy.

## Supplementary Information

**Additional file 1:** **Supplementary method.** **Supplementary Figure 1.** ASDR of T2DM attributable to non-high BMI and high BMI for 21 regions, in 2019.

**Additional file 2: Supplementary Table 1.** Numbers deaths of T2DM attributable to nonhigh BMI and high BMI and their percentage changes. **Supplementary Table 2.** Number DALYs of T2DM attributable to nonhigh BMI and high BMI and their percentage changes. **Supplementary Table 3.** ASMR of T2DM attributable to nonhigh BMI and high BMI and EAPC. **Supplementary Table 4.** ASDR of T2DM attributable to nonhigh BMI and high BMI, and EAPC.

## Data Availability

The dataset(s) supporting the conclusions of this article are available in the Global Health Data Exchange repository, https://vizhub.healthdata.org/gbd-results/.

## Abbreviations

T2DM: Type 2 diabetes mellitus
BMI: Body mass index
EAPC: Estimated annual percentage change
DALYs: Disability-adjusted life years
SDI: Socio-demographic index
GBD: Global burden of disease
GHDEx: Global health data exchange
ASR: Age-standardized rate
ASDR: Age-standardized disability-adjusted life-year rate
ASMR: Age-standardized mortality rate
YLDs: Years lived with disability
YLLs: Years of life lost
CI: Confidence interval
NAFLD: Non-alcoholic fatty liver disease
GATHER: Guidelines for accurate and transparent health estimates reporting
MetS: Metabolic syndrome
UI: Uncertain interval
