# Supplementary material for: Global burden of type 2 diabetes attributable to non-high body mass index from 1990 to 2019
Source: BMC Public Health. 2023 Jul 12;23:1338. doi: 10.1186/s12889-023-15585-z (PMC10337097; doi:10.1186/s12889-023-15585-z)
Supplement: Supplementary file 1 — Additional file 1: Supplementary method. Supplementary Figure 1. ASDR of T2DM attributable to non-high BMI and high BMI for 21 regions, in 2019. [file 12889_2023_15585_MOESM1_ESM.docx]

**Supplementary meterial**

1. **Supplementary method**
   1. **Definitions**

Type 2 diabetes mellitus (T2DM) was defined by the presence of any of the following: (a) fasting plasma glucose ≥126 mg/dL (7 mmol/L); (b) reporting to be on drug or insulin treatment for T2DM, and/or (c) with the International Classification of Diseases (ICD) version 10 codes: E11-E11.1, E11.3-E11.9 [1]. High body mass index (BMI) was defined as BMI > 25 kg/m^2^ in adults (ages 20+) and using thresholds based on the International Obesity Task Force standards for patients aged 1-19 years [2].

- 1. **Population Attributable Fractions**

The attributable proportions of age-standardized mortality rate (ASMR) and age-standardized disability-adjusted life-year rate (ASDR) attributable to high BMI were evaluated using population attributable fractions (PAFs), which were derived using a previously established comparative risk assessment approach. The PAFs represent the ASMR or ASDR that might have been avoided if the exposure to high BMI had been lowered to an optimal exposure situation [3]. The estimates of death and disability-adjusted life-years (DALYs) attributable to high BMI and all risk factors were obtained from the Global Health Data Exchange GBD Results Tool (<https://vizhub.healthdata.org/gbd-results/>). The burdens of T2DM attributable to non-high BMI were defined as the burden of T2DM attributable to all risk factors minus the burden of T2DM due to high BMI to quantify the attributable burdens of T2DM attributable to non-high BMI.

- 1. **Age-standardized Rate**

Age-standardization is a essential and representative step when comparing multiple population with different age structures or for the same population over time in which the age profiles change accordingly. The age-standardized rate (ASR) (per 100,000 population) was calculated by the world standard population developed for the global burden of disease (GBD) study. The formula can be obtained as follows: $ASR=\frac{\sum_{i=1}^{A} a_{i}w_{i}}{\sum_{i=1}^{A} w_{i}}\times100,000$, $a_{i}$ means the age-specific rate in the $i^{th}$ age subgroup, $w_{i}$ denotes the number of persons (or weight) in the same age subgroup $i$ of the chosen reference standard population, and $A$ means the number of age groups [4]. Based on the formula, we calculated the ASMR and ASDR to reduce the potential confounding effect of age and to examine geographic variations.

- 1. **Socio-demographic Index (SDI)**

The Socio-demographic Index (SDI) is a composite indicator of a geographical location’s development status created by GBD research team. SDI was calculated as the geometric mean of total fertility rates among female under the age of 25 (TFU25), mean educational attainment for individuals aged 15 years and older (EDU15+), and lag distributed income per capita (LDI). The TFU25, EDU15+, and LDI were calculated by the formula as follows: $I_{cly}=max\left( \frac{C_{ly}-C_{low}}{C_{high}-C_{low}},0.005 \right)$. $I_{cly}$ represent as the conversion value of covariate $C$ in "0 - 1" scale in region $l$ and year $y$. $C_{cly}$*,* $C_{min}$, and $C_{max}$ denote measured values, minimum values, and maximum values of component $C$ in region $l$ and year $y$, respectively. The value of the upper and lower bound for each input index were given by GBD2017 [5]. Due to the negative relationship between TFU25 and development, ${I_{TFU25}=1-I}_{TFU25ly}$ was used as the conversion value of "0 - 1". The geometric mean of these three indices is: $SDI=\sqrt[3]{I_{LSD}\times I_{EDU15}\times I_{TFU25}}$. The scale of SDI was from 0 to 1, where 0 represents the lowest level of development, and 1 represents the highest level of development [6]. The SDI in 204 countries and territories from 1990 to 2019 were collected from GBD 2019. In GBD 2019, the 204 countries and regions were not only divided into five groups based on the SDI, but also divided into 21 GBD regions based on geographical location. In this study, we used SDI to determine the relationship between the development level of a region or country and non-high BMI related T2DM burden (ASMR and ASDR).

- 1. **References**

1. **Global burden of 369 diseases and injuries in 204 countries and territories, 1990-2019: a systematic analysis for the Global Burden of Disease Study 2019**. *Lancet* 2020, **396**(10258):1204-1222.

2. **Global burden of 87 risk factors in 204 countries and territories, 1990-2019: a systematic analysis for the Global Burden of Disease Study 2019**. *Lancet* 2020, **396**(10258):1223-1249.

3. **Global, regional, and national comparative risk assessment of 84 behavioural, environmental and occupational, and metabolic risks or clusters of risks for 195 countries and territories, 1990-2017: a systematic analysis for the Global Burden of Disease Study 2017**. *Lancet* 2018, **392**(10159):1923-1994.

4. Liu Z, Jiang Y, Yuan H, Fang Q, Cai N, Suo C, Jin L, Zhang T, Chen X: **The trends in incidence of primary liver cancer caused by specific etiologies: Results from the Global Burden of Disease Study 2016 and implications for liver cancer prevention**. *J Hepatol* 2019, **70**(4):674-683.

5. **Global, regional, and national incidence, prevalence, and years lived with disability for 354 diseases and injuries for 195 countries and territories, 1990-2017: a systematic analysis for the Global Burden of Disease Study 2017**. *Lancet* 2018, **392**(10159):1789-1858.

6. **Global age-sex-specific fertility, mortality, healthy life expectancy (HALE), and population estimates in 204 countries and territories, 1950-2019: a comprehensive demographic analysis for the Global Burden of Disease Study 2019**. *Lancet* 2020, **396**(10258):1160-1203.

1. **Supplementary Figure**
   1. **Supplementary Figure 1. ASDR of T2DM attributable to non-high BMI and high BMI for 21 regions, in 2019.**

**
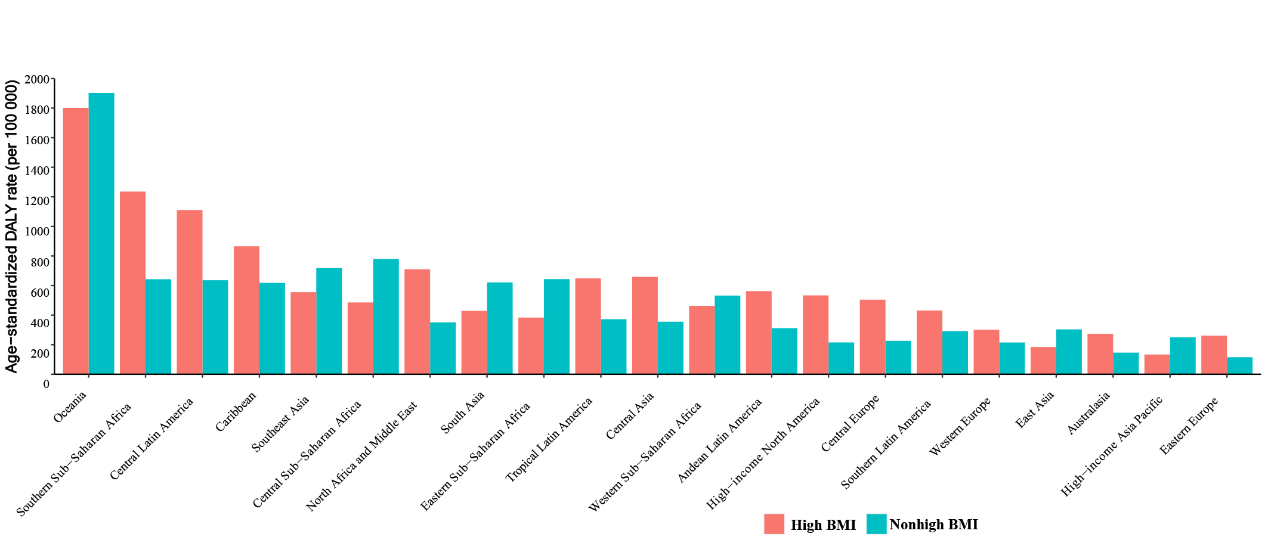
**

**Abbreviations:** ASDR, Age-standardized disability-adjusted life-year rate; T2DM, type 2 diabetes mellitus; BMI, body mass index.
